# Prediction of prognosis and treatment effect of cervical cancer by immunogenic cell death-related genes

**DOI:** 10.7717/peerj.21635

**Published:** 2026-10-07

**Authors:** Xiufang Li, Xiaona Luan, Yewu Sun, Liying Huang, Yan Kong, Tian Tian

**Affiliations:** 1 Department of Gynecology, Jiaozhou Hospital Affiliated to Dongfang Hospital of Tongji University, Qingdao, Shandong Province, China; 2 Health Care Department, Qingdao Municipal Hospital, Qingdao, Shandong Province, China; 3 Department of Gynecology, The Affiliated Hospital of Qingdao University, Qingdao, Shandong Province, China

**Keywords:** Uterine cervical neoplasms, Immunogenic cell death, Prognosis, Immunotherapy

## Abstract

**Background:**

Immunogenic cell death (ICD) is a potential mechanism that mediates adaptive immune responses during anticancer therapy. However, expression patterns and clinical implications of ICD related genes in cervical squamous cell carcinoma and endocervical adenocarcinoma (CESC) remain unclear. This study aimed to investigate the associations of 34 ICD related genes with immune characteristics, prognosis, and immune checkpoint blocking (ICB) efficacy in CESC and to predict drug responses across different risk subgroups.

**Methods:**

Ribonucleic acid sequencing (RNA-seq) transcriptomic profiles, standard normalized variate (SNV) data, and corresponding clinicopathological information were retrieved from The Cancer Genome Atlas (TCGA). Consensus clustering was applied to identify two ICD related molecular subtypes. A prognostic signature was constructed using least absolute shrinkage and selection operator (LASSO) Cox regression and validated using immunohistochemistry (IHC) and receiver operating characteristic (ROC) curve analyses. Immunotherapy responses were predicted based on the risk score of the established model.

**Results:**

The ICD-high subtype (C2) was associated with favorable clinical outcomes and enhanced immune activation. The prognostic signature comprised six genes: ATG5, FOXP3, IFNG, IL1B, PDIA3, and TNF. IHC confirmed higher expression of ATG5 and PDIA3 in cervical cancer (CC) tissues than in normal cervical tissues, and ROC curves verified the predictive accuracy. Furthermore, immunophenoscore (IPS) and tumor immune dysfunction and exclusion (TIDE) scores suggested that low risk patients exhibited better responses to anti programmed cell death protein-1 (PD-1) immunotherapy.

**Conclusions:**

This study characterized the expression profiles of ICD related genes and established a novel ICD based classification system for CESC. Comprehensive analyses of the tumor immune microenvironment further validated the capacity of this signature in predicting prognosis and immunotherapy benefits, which may help identify CC patients who are most likely to benefit from immunotherapy.

## Introduction

Cervical cancer (CC) is the fourth most frequently diagnosed cancer and the fourth leading cause of cancer-related mortality among women worldwide (Bray et al., 2024). In recent years, immunotherapy targeting cytotoxic T-lymphocyte-associated protein-4 (CTLA-4), programmed cell death protein-1 (PD-1), and programmed cell death ligand 1 (PD-L1) has revolutionized the treatment landscape for multiple malignancies (Huang et al., 2022). In June 2018, the U.S. Food and Drug Administration (FDA), based on preliminary results from the phase II KEYNOTE-158 trial, the FDA approved pembrolizumab for recurrent or metastatic CC (Borcoman & Le Tourneau, 2020; Chung et al., 2018). Nevertheless, reliable biomarkers for stratifying patients and tailoring immunotherapy remain lacking in CC.

Immunogenic cell death (ICD) is a form of regulated cell death (RCD) that elicits adaptive immune responses by releasing damage associated molecular patterns (DAMPs), including high mobility group box 1 (HMGB1), extracellular adenosine triphosphate (ATP), and surface exposed calreticulin (CRT) (Ahmed & Tait, 2020). Mounting evidence indicates that ICD plays a pivotal role in triggering antitumor immune responses. In this study, we aimed to characterize the associations between ICD related biomarkers and immune features, prognosis, and immunotherapy responses in carcinoma and endocervical adenocarcinoma (CESC). We identified an ICD related gene signature that may serve as a reliable indicator for predicting clinical outcomes and immunotherapy benefits in patients with CESC.

## Materials and Methods

For the training set, ribonucleic acid sequencing (RNA-seq) transcriptomic information, mutation annotation format-type single nucleotide variants (SNVs) data, and matching clinicopathological data were obtained from The Cancer Genome Atlas (TCGA) (http://portal.gdc.cancer-gov/), which included 309 samples (normal count: 3; tumor count: 306). We downloaded the prognostic information of 300 patients with CC from the Gene Expression Omnibus (GEO) (login number: GSE44001; https://www.ncbi.nlm.nih.gov/geo/query/acc.cgi). The expression levels of ICD-related genes were extracted by “limma” package. The ICD-related genes were derived from Garg Ads study (Garg, De Ruysscher & Agostinis, 2016). Somatic mutation data were obtained from TCGA Genomic Data Commons (GDC) portal website (https://portal.gdc.cancer.gov/). We used the “maftools” package (Version: 4.1.2; R Core Team, 2021) to create a waterfall map depicting the genetic mutations in patients in the high- and low-expression groups. The top 20 genes with the highest mutation frequencies were selected for visualization. The overall framework of the article is shown in Fig. 1.

**Figure 1:**
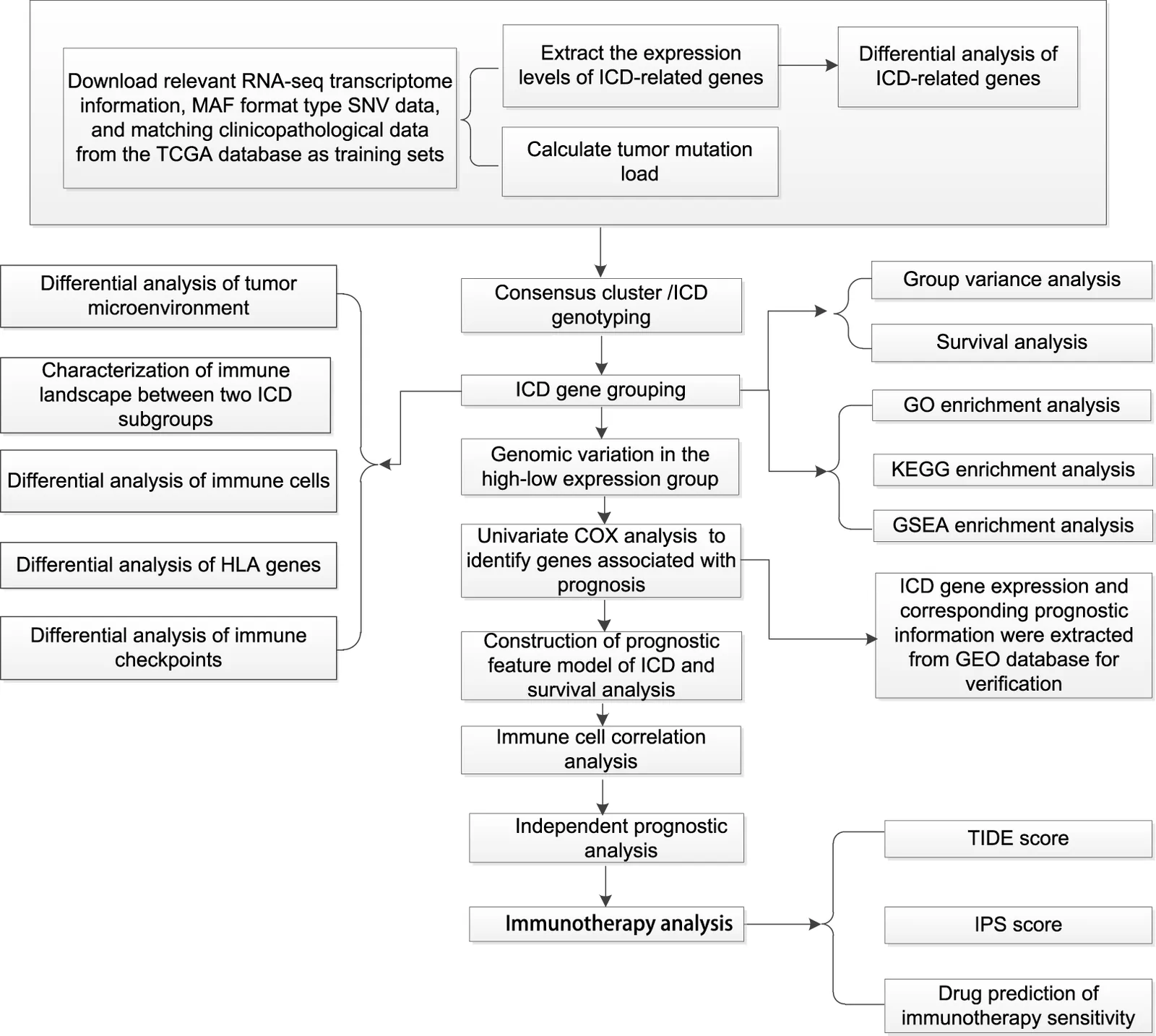
The flow chart of the research process.

### Identification of differentially expressed genes

Wilcoxon rank-sum test was performed to screen ICD-related differentially expressed genes (DEGs), with the screening thresholds defined as false discovery rate (FDR) < 0.05 and |log_2_ (fold change)| > 2. The expression patterns of DEGs were visualized *via* the R package pheatmap (version 4.1.3; R Core Team, 2022a).

### Consensus clustering and ICD subtyping

Univariate Cox regression analysis was carried out with the R packages limma and survival (version 4.1.3; R Core Team, 2022a) to screen ICD genes correlated with patient prognosis. Consensus clustering was implemented on the expression profiles of ICD-related genes to distinguish distinct molecular subtypes. All samples were stratified into low-ICD (C1) and high-ICD (C2) subgroups based on the median expression value, and Kaplan–Meier survival curves were plotted accordingly. Survival comparisons were further conducted for subgroups stratified by tumor mutational burden (TMB) alone, as well as combined TMB and risk score subgroups (Bersanelli, 2020; Marabelle et al., 2020).

### Functional enrichment analyses

Gene Ontology (GO) and Kyoto Encyclopedia of Genes and Genomes (KEGG) enrichment analyses were conducted using the clusterProfiler package (version 4.2.2; R Core Team, 2022b). Gene set enrichment analysis (GSEA) was performed through the MSigDB database to compare pathway enrichment between C1 and C2 subtypes, with the top 30 pathways displayed.

### Immune microenvironment characterization

Tumor microenvironment (TME) scores, including immune score, stromal score, and tumor purity score, were calculated using the estimate and limma packages (version 4.1.3; R Core Team, 2022a). The CIBERSORT algorithm was applied to estimate the infiltration levels of 22 immune cell types. Associations between ICD subtypes and immune functions were evaluated by comparing immune cell infiltration, human leukocyte antigen (HLA) family genes, and immune checkpoint related gene expression.

### Construction and validation of the ICD prognostic signature

Prognostic ICD genes were screened using univariate Cox regression. Least absolute shrinkage and selection operator (LASSO) Cox regression was performed using the glmnet package (version 4.1.3; R Core Team, 2022a) to establish a prognostic signature, with the optimal model determined by the lowest Akaike information criterion (AIC) (Ikemoto et al., 2023). Patients were stratified into high and low risk groups by the median risk score in the TCGA cohort. The GSE44001 dataset was used as an external validation cohort. Prognostic performance was assessed using time dependent receiver operating characteristic curves and Kaplan Meier survival analysis. Univariate and multivariate Cox regression analyses were conducted to verify whether the risk score was an independent prognostic factor. Spearman correlation analysis was used to examine the relationship between risk score and immune cell infiltration.

### Immunohistochemical validation

immunohistochemistry (IHC) was performed to validate the expression of ATG5 and PDIA3 in five pairs of CC and normal cervical tissues obtained from the Affiliated Hospital of Qingdao University. The study was approved by the Ethics Committee of the Affiliated Hospital of Qingdao University (approval No.: QYFY WZLL 28550). All patients enrolled in this study provided written informed consent, and each participant signed a confidentiality agreement. IHC staining was performed on paraffin-embedded normal control tissues (A, C) and cancer tissues (B, D). After dewaxing, heat-induced antigen retrieval was conducted on tissue sections. The sections were incubated overnight at 4 °C with primary antibodies against ATG5 (Cat# 10181-2-AP, RRID: AB_10694400; Proteintech, Rosemont, IL, USA) or PDIA3 (Cat# 15967-1-AP, RRID: AB_2881232; Proteintech, Rosemont, IL, USA). All images were captured at 400 × magnification, and brown cytoplasmic staining indicated positive expression of target proteins.

### Immunotherapy and chemotherapy prediction

Immunophenoscore (IPS) was obtained from the The Cancer Immunome Database (TCIA) database (https://tcia.at/home). Tumor immune dysfunction and exclusion (TIDE; http://tide.dfci.harvard.edu/) was used to predict immunotherapy responses (Jiang et al., 2018). The pRRophetic package (R Core Team, 2014) was applied to estimate the half maximal inhibitory concentration (IC_50_) of common chemotherapeutic agents. Spearman correlation analysis was used to assess the relationship between IC_50_ and ICD risk score.

## Results

### Two ICD-related subtypes identified by consensus clustering

Multiple ICD-related genes including CASP8, MYD88, CALR, HSP90AA1, IL10, FOXP3, IFNG, BAX and TNF were upregulated in CESC tissues (Fig. 2). Consensus clustering stratified CC samples into two stable molecular subtypes: the C2 and the C1 (Figs. 3A–3C). Survival analysis demonstrated that patients with the C2 exhibited a more favorable prognosis, while those with the C1 subtype had poorer clinical outcomes. In addition, high TMB was correlated with longer overall survival. Patients with both high ICD-related gene expression and high TMB presented the most favorable prognosis (Figs. 3D–3F).

**Figure 2:**
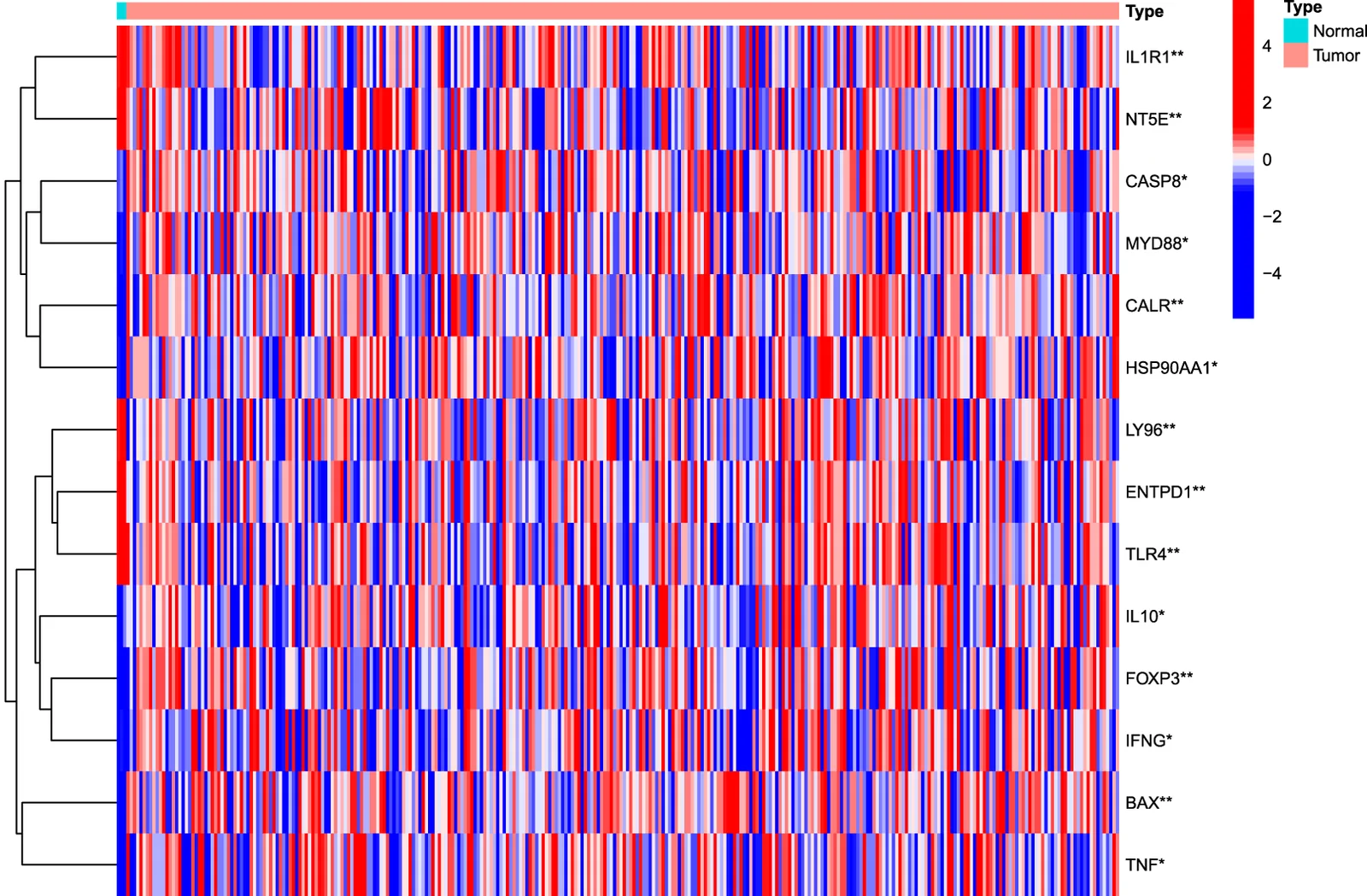
Differential expression of ICD-related genes in normal and tumor samples. The horizontal axis corresponds to individual samples, while the vertical axis represents ICD-related genes. IL1R1, NT5E, YL96, ENTPD1 and TLR4 are significantly upregulated in normal tissues. In contrast, the expression of CASP8, MYD88, CALR, HSP90AA1, IL10, FOXP3, IFNG, BAX and TNF is markedly elevated in tumor samples. *P < 0.05, and **P < 0.01.

**Figure 3:**
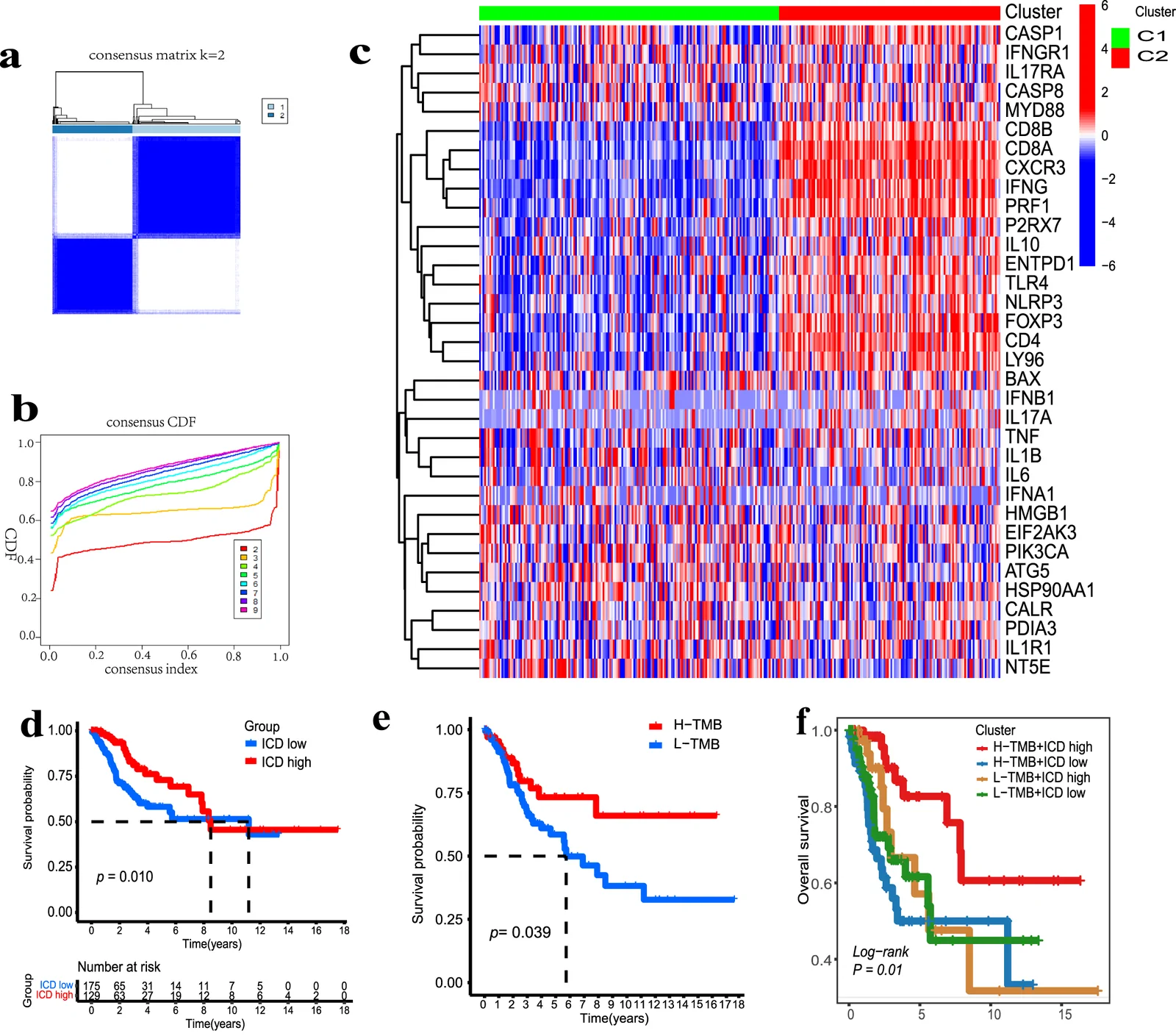
The result of cluster grouping. (A and B) Samples were classified into distinct subgroups *via* consistent clustering based on the expression levels of ICD-related genes. (C) The horizontal axis indicates individual samples, and the vertical axis represents the expression levels of ICD-related genes. Blue denotes low gene expression, whereas red indicates high expression. Cluster 1 was defined as the ICD low-expression group, and Cluster 2 as the ICD high-expression group. (D) The ICD high-expression group exhibited a more favorable prognosis than the ICD low-expression group. (E) Patients in the high TMB group showed better prognostic outcomes compared with those in the low TMB group. (F) Combined survival analysis revealed that patients with both high TMB and high ICD expression had the best prognosis, while the combination of low TMB and low ICD expression correlated with the worst clinical prognosis.

### The result of functional and pathway enrichment analysis

DEGs in the ICD high subtype were significantly enriched in immune related biological processes, including leukocyte mediated immunity, lymphocyte activation, immunoglobulin production, antigen binding, and immune receptor activity (Figs. 4A, 4B). GSEA further confirmed that the ICD high subtype was prominently enriched in immune related pathways, such as adaptive immune response, T cell activation and differentiation, antigen processing and presentation, chemokine signaling, and cytokine cytokine receptor interaction (Figs. 4C, 4D).

**Figure 4:**
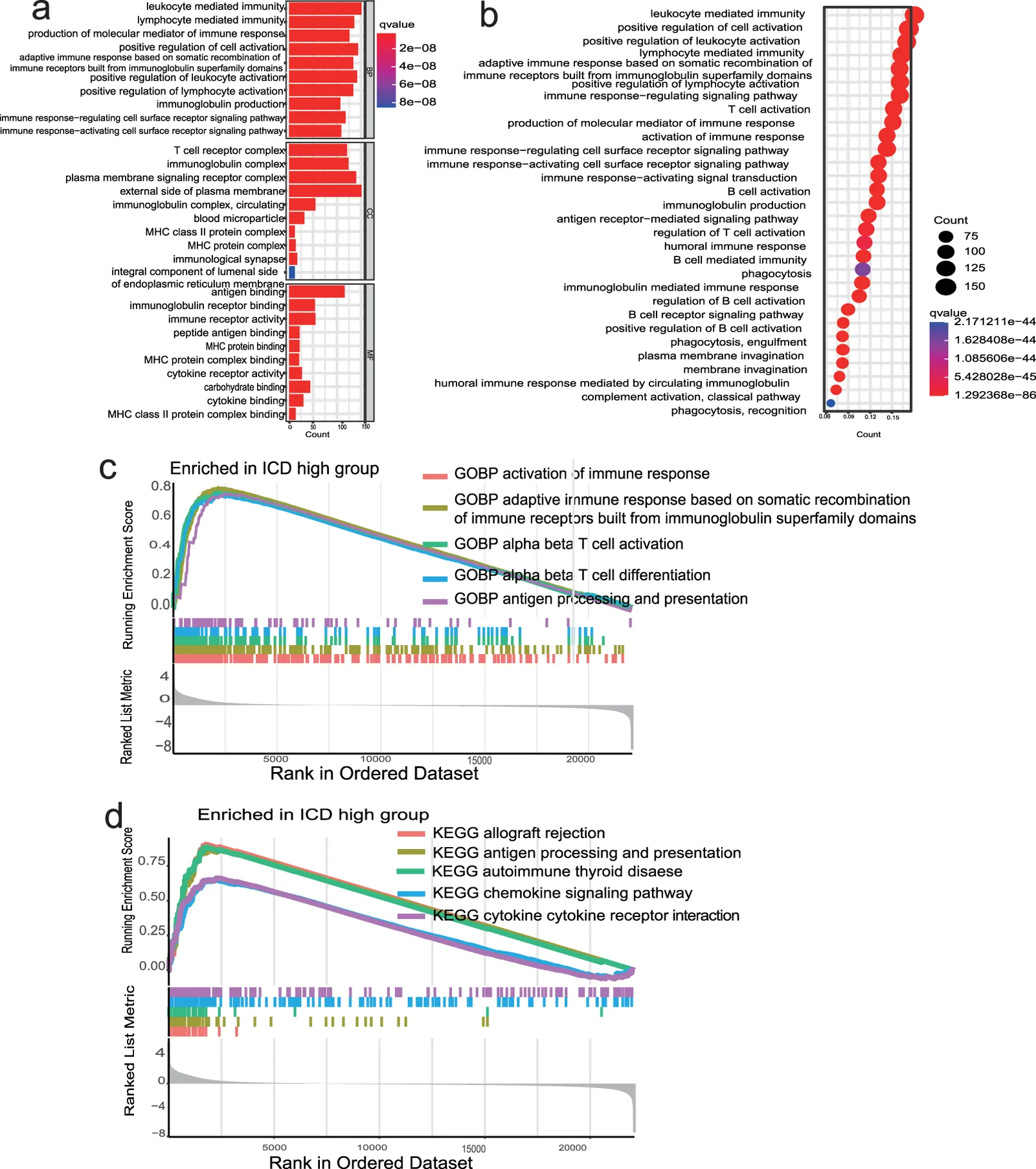
Functional and pathway enrichment analysis. (A) The results of GO enrichment analysis. The vertical axis lists pathway terms and the horizontal axis indicates the gene count enriched in each pathway. The column color reflects the enrichment significance; a redder color suggests a more significant enrichment of differentially expressed genes. (B) The KEGG enrichment analysis results. The vertical axis corresponds to pathway names and the horizontal axis represents the enrichment ratio of genes in each pathway. The circle size indicates the enriched gene number, while the circle color denotes enrichment significance. Likewise, deeper red indicates higher enrichment significance of differential genes. (C and D) The enriched functional pathways identified in the ICD high-expression group.

### Somatic mutation profiles of ICD subtypes

We noted a distinct spectrum of somatic mutants among these subtypes. Although TTN, PIK3CA, KMT2C, MUC1, and KMT2 were the most common mutations, the relative frequencies of different subtypes varied. The ICD-high group showed high mutation frequencies of TTN and PIK3CA, accounting for 37% and 30% of the total, respectively, whereas the ICD-low group accounted for only 23% and 27%, respectively (Fig. 5).

**Figure 5:**
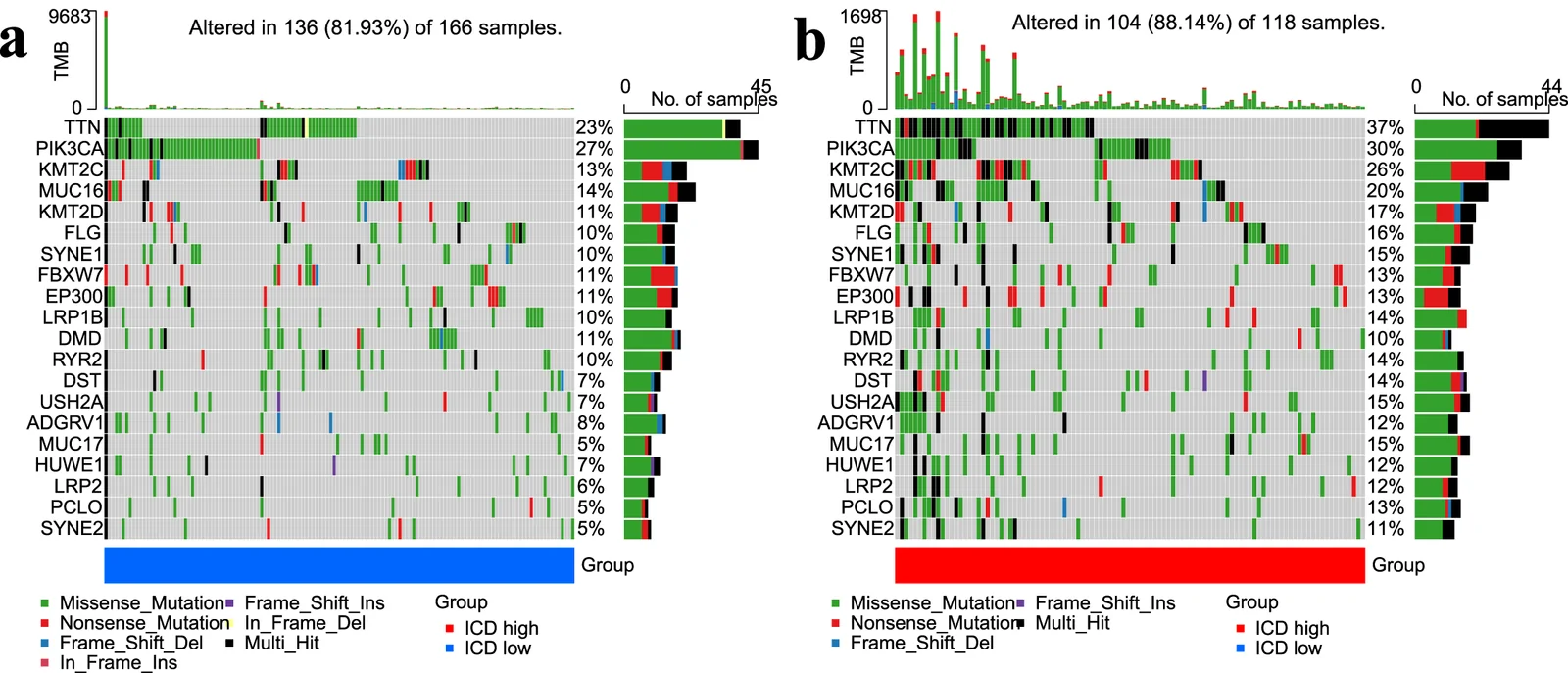
(A and B) Comparison of somatic mutations among different subtypes of ICDs. The waterfall plot illustrates the mutation profiles of patients in the ICD low-expression and high-expression groups. The horizontal axis represents individual samples and the vertical axis denotes mutant genes. Different colors correspond to various mutation types. Meanwhile, the mutation frequency of each gene can be clearly visualized in this plot.

### Immune microenvironment features of ICD subtypes

The ICD high subtype showed significantly higher immune scores and lower tumor purity than the ICD low subtype, indicating enhanced immune cell infiltration (Fig. 6). CIBERSORT analysis demonstrated that the ICD high subtype was characterized by increased infiltration of CD8^+^ T cells, CD4^+^ memory activated T cells, follicular helper T cells, M1 macrophages, and resting dendritic cells (DCs). In contrast, the ICD low subtype showed higher proportions of CD4^+^ memory resting T cells, M0 macrophages, activated DCs, activated mast cells, and eosinophils (Figs. 7A, 7B). Most HLA family and immune checkpoint genes were upregulated in the ICD high subtype (Figs. 7C–7E), suggesting that ICD-high tumors correspond to immunologically “hot tumors” with heightened sensitivity to immune checkpoint inhibitors (ICIs).

**Figure 6:**
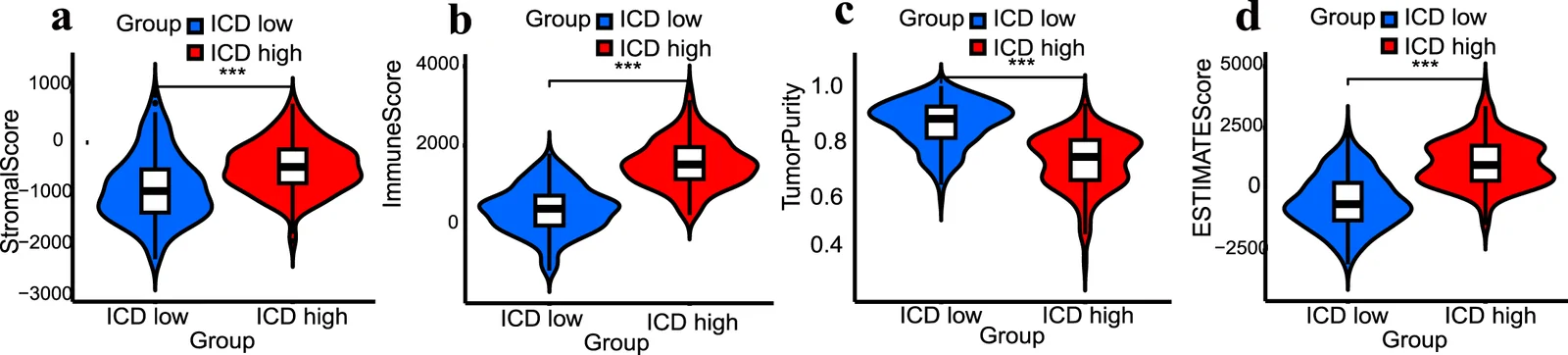
Differential analysis of tumor immune microenvironment. The abscissa represents the group of ICDs and the ordinate represents the tumor microenvironment score. (A, B, D) The scores of stromal cells, immune cells, and comprehensive were higher in the ICD high expression group. (C) Low ICD expression group had a higher tumor purity score. ***P < 0.001.

**Figure 7:**
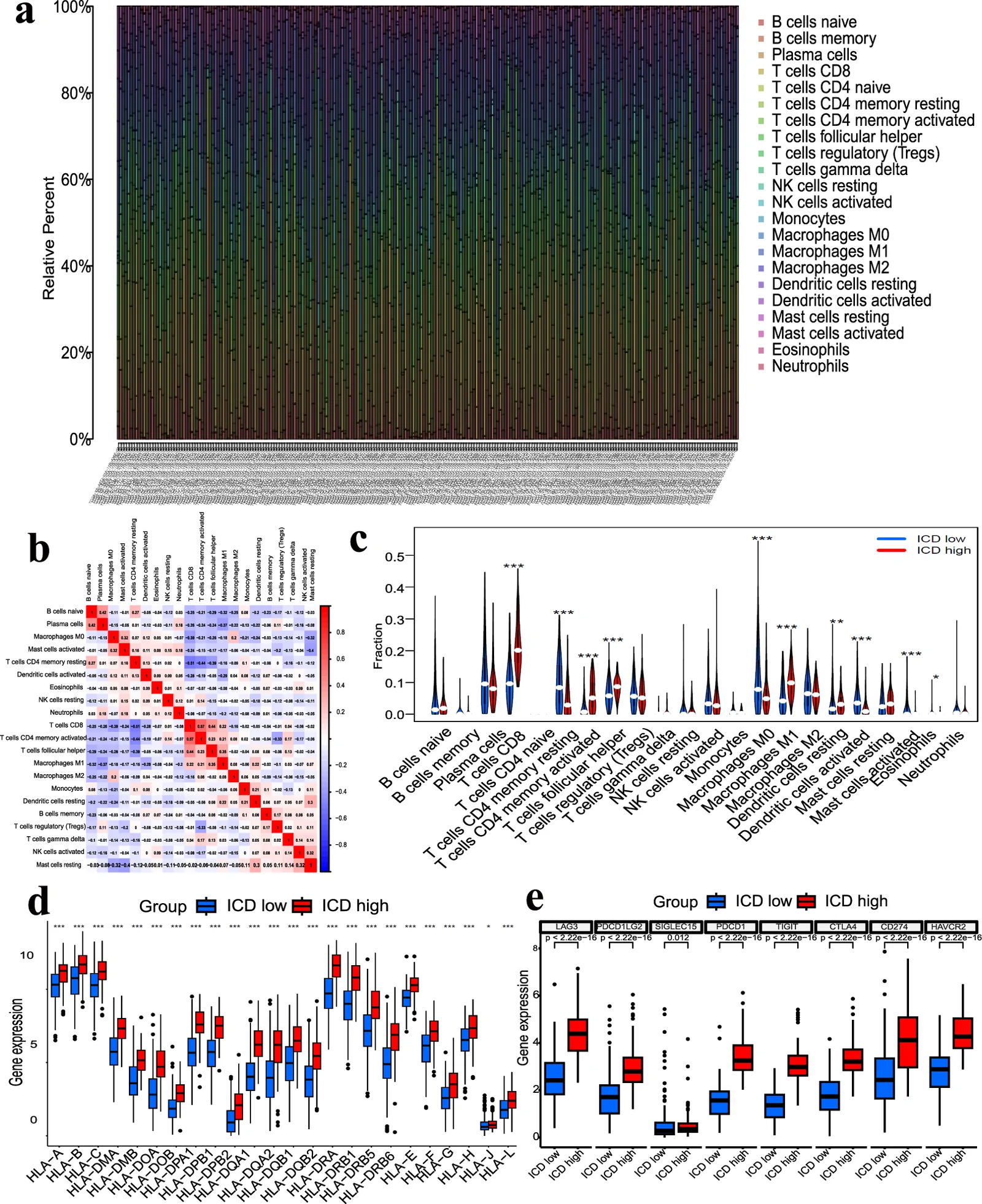
Analysis of immune characteristics. (A) The abscissa is the name of the sample, the vertical axis is the number of immune cells. (B) The abscissa and the vertical axis are the names of the immune cells, and the numbers inside are the correlation coefficients. Blue means negative correlation, red means positive correlation. (C) The horizontal axis represents the name of immune cells, the vertical axis represents the number of immune cells, the blue is the group with low ICD expression, and the red is the group with high ICD expression. (D) The horizontal axis represents the HLA-related genes and the vertical axis represents the gene expression, with blue representing the group with low ICD expression and red representing the group with high ICD expression. (E) On the horizontal axis are genes associated with immune checkpoints, and on the vertical axis are gene expression levels, with low ICD expression in blue and high ICD expression in red. *P < 0.05, **P < 0.01, and ***P < 0.001.

### Construction and validation of the ICD prognostic signature

Univariate Cox regression identified 11 ICD related genes significantly associated with overall survival (Fig. 8A). LASSO regression was used to establish a six gene prognostic signature: ATG5, FOXP3, IFNG, IL1B, PDIA3, and TNF. The risk score was calculated as follows: Risk score = ATG5 × 0.2147 + FOXP3 × (−0.3920) + IFNG × (−0.1026) + IL1B × 0.2072 + PDIA3 × 0.3367 + TNF × 0.1178. In the TCGA training cohort, the area under the ROC curve (AUC) values for 1, 3, and 5 year survival were 0.814, 0.702, and 0.721, respectively (Fig. 8D). In the GEO validation cohort, the 1, 3, and 5 year AUC values were 0.636, 0.663, and 0.562, respectively (Fig. 8E). In the TCGA group, the low-risk group had a better prognosis (Fig. 8G), and this was also confirmed in the GEO database (Fig. 8H). The low-risk group showed significantly better overall survival than the high-risk group in TCGA cohorts (Fig. 8F). Univariate and multivariate Cox regression confirmed that the ICD risk score was an independent prognostic factor for CESC (Figs. 8I, 8J). IHC verified that ATG5 and PDIA3 were overexpressed in CC tissues compared with normal tissues (Figs. 9A–9D).

**Figure 8:**
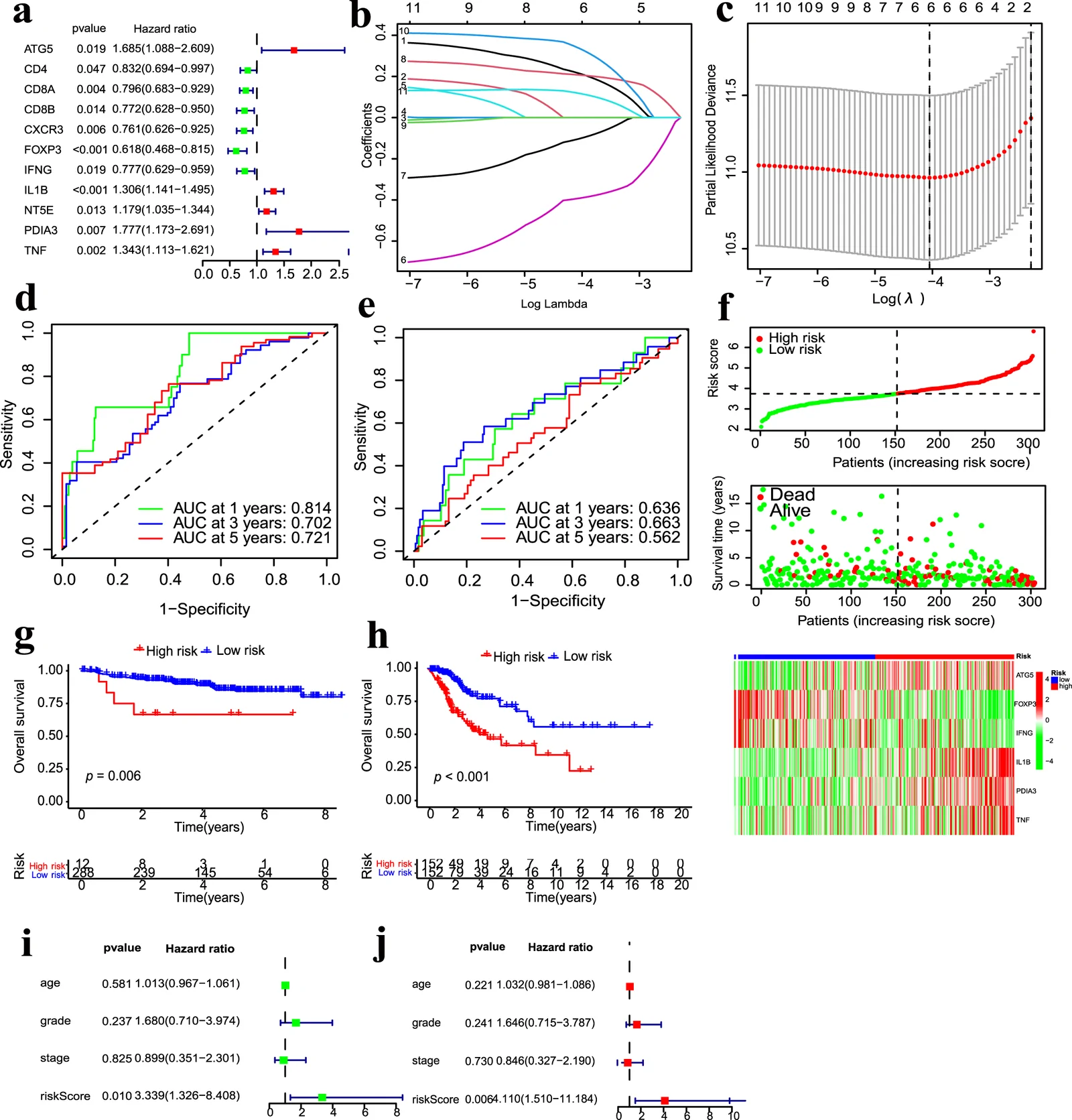
Model construction and verification. (A) UniCox analysis was performed to identify prognostic genes: Visualization of HR values in forest map, green represents low-risk genes, red represents high-risk genes. (B) The graph of lasso regression. (C) The graph of cross validation. (D), (E) ROC curves corresponding to prediction models in TCGA database (G) and GEO database (H). TCGA verification & GEO verification based on the patient’s risk score, the sample was divided into two groups: high and low risk, with low risk shown in blue and high risk in red. The abscissa is the survival time and the ordinate is the survival rate. (F) Risk curve: the abscissa represents the sorted samples. Risk curve: the vertical axis represents the risk score. The risk to patients increases from left to right. According to the median risk score of the patients, the sample was divided into high- and low-risk groups, with the low-risk group represented in green and the high-risk group represented in red. In the small graph survival state, the ordinate represents the survival time. The points in the graph are shown in red for patients who died and in green for those who survived. As the risk to patients increases, the risk of death also increases, ATG5, IL-1B, PDIA3, and TNF were high-risk genes, while FOXP3 and IFNG were low-risk genes. (I) Univariate COX regression analysis: risk score is correlated with patient outcome. (J) Multivariate COX regression analysis showed that risk scores could be used as independent prognostic predictor.

**Figure 9:**
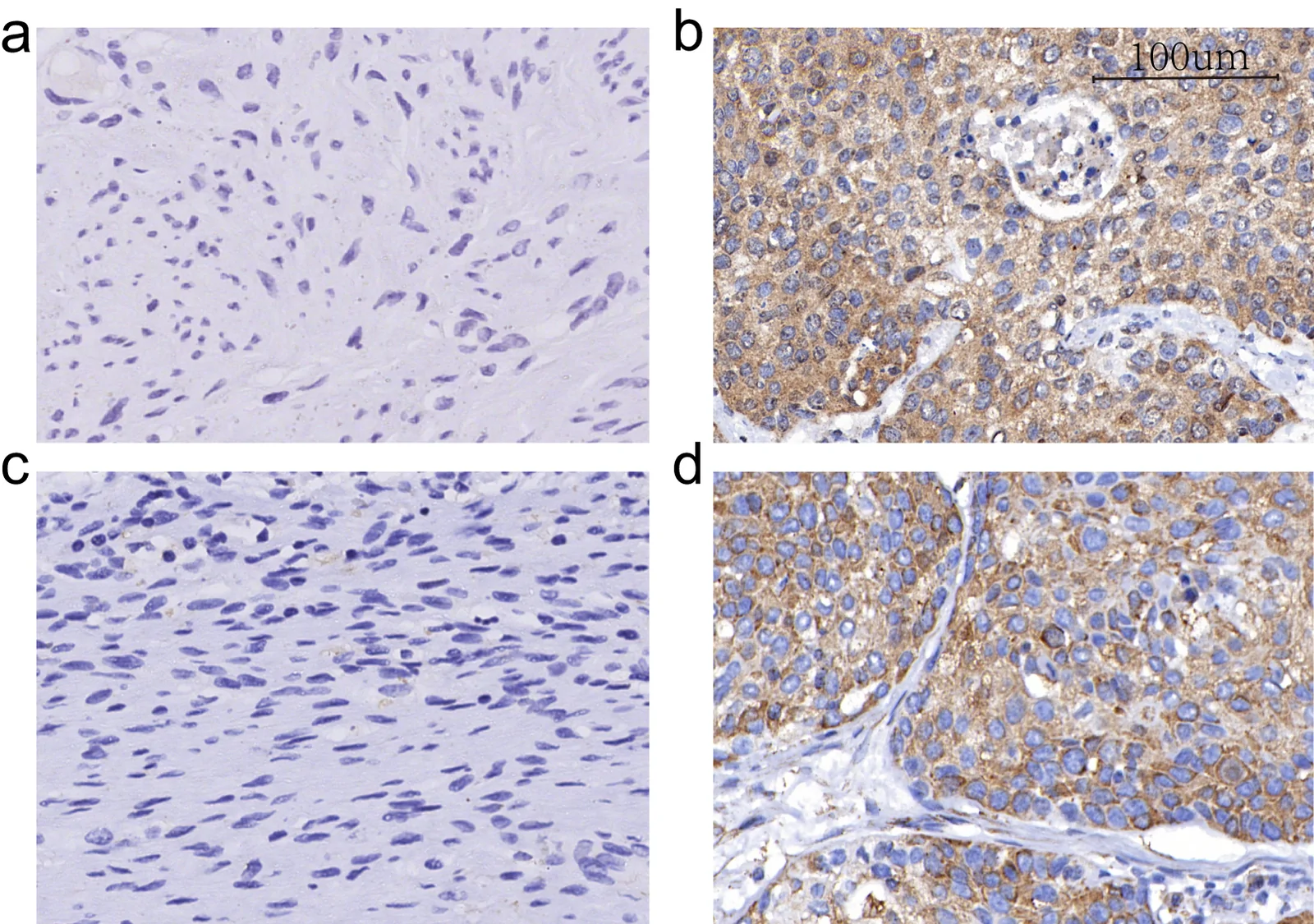
Immunohistochemistry verified the expression of ATG5 and PDIA3 in normal tissues and CC tissues. (A) ATG5 expression in normal cervical tissues. (B) ATG5 expression in CC. (C) PDIA3 expression in normal cervical tissues. (D) PDIA3 expression in CC.

### Associations between ICD risk score and immune microenvironment

High risk scores were positively correlated with activated DCs, M0 macrophages, resting mast cells, neutrophils, resting NK cells, and CD4^+^ memory resting T cells. In contrast, high risk scores were negatively correlated with B cells, resting DCs, M1 macrophages, CD8^+^ T cells, follicular helper T cells, and regulatory T cells (Tregs; Fig. 10).

**Figure 10:**
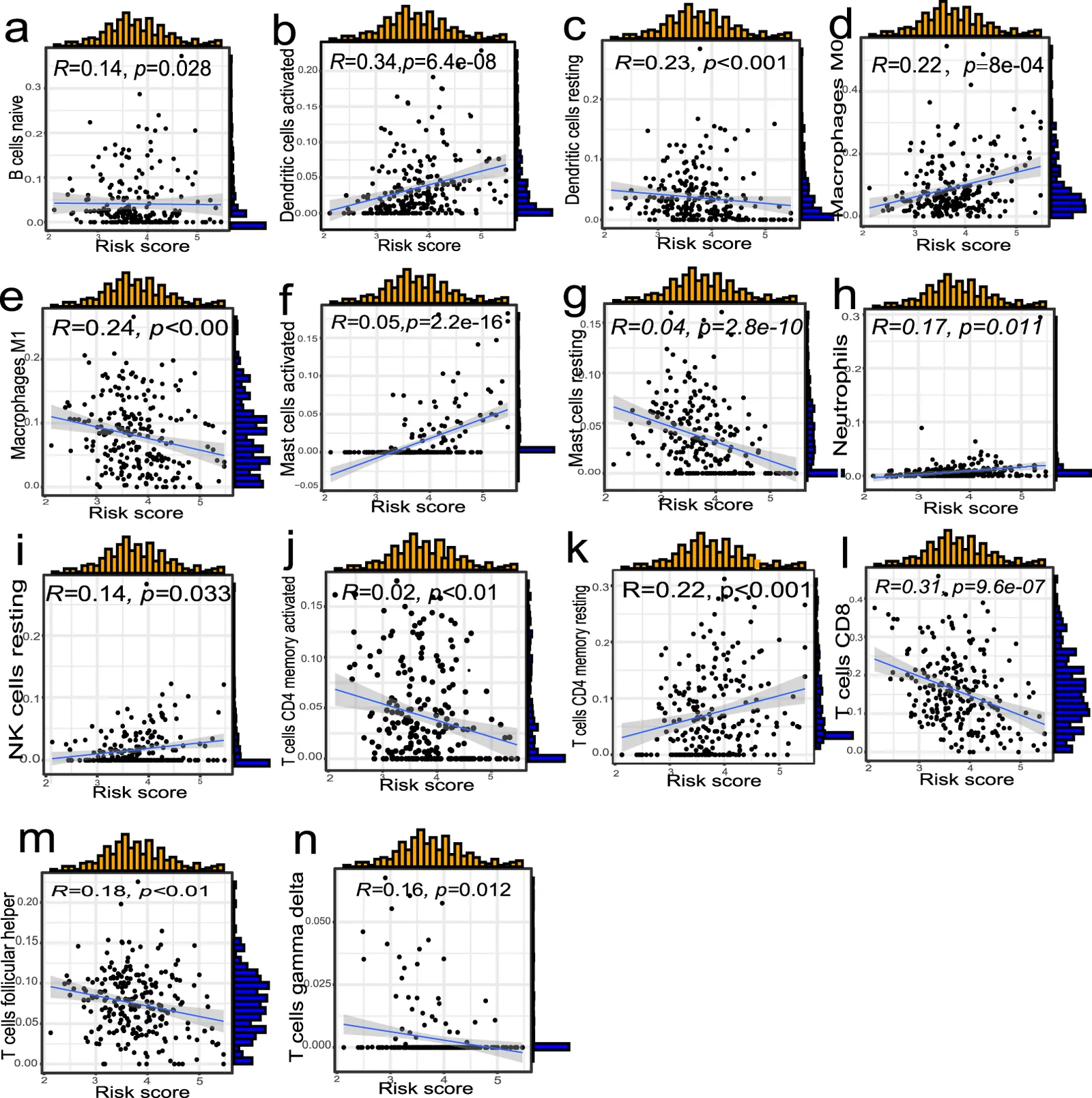
(A–N) Correlation analysis between risk score and immune cells. The X-axis represents the risk score, the y-axis represents the number of immune cells, and the dots represent the samples.

### Immunotherapy and chemotherapy prediction

Low risk patients exhibited higher IPS, regardless of CTLA 4 expression status, indicating superior responses to anti PD-1 immunotherapy (Figs. 11A–11D). TIDE scores were significantly lower in the low-risk group, suggesting reduced immune escape and better immunotherapy efficacy (Fig. 11E). Chemotherapy sensitivity analysis revealed that high risk patients might be more sensitive to bosutinib, camptothecin, lenalidomide, methotrexate, nilotinib, rapamycin, sunitinib, vinblastine, and other agents, whereas low risk patients showed higher sensitivity to bexarotene, cytarabine, and imatinib (Table 1).

**Figure 11:**
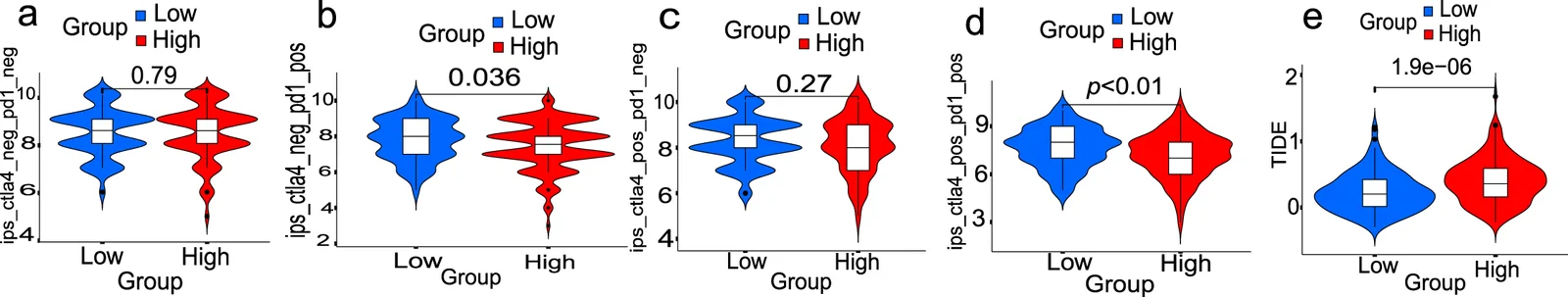
Immunotherapy analysis. (A–D) Immunotherapy analysis-TCIA. (B), (D) The IPS score of the low-risk group was higher, indicating that the low-risk group had a better effect on PD-1 treatment. (E) Immunotherapy analysis-TIDE. The TIDE score was higher in the high-risk group, indicating that immunotherapy was more effective in this group.

**Table 1:** The corresponding drugs and *P* values in the high-low risk group.

Drug names corresponding to low IC_50_ in high-risk group	*P*	Drug names corresponding to low IC_50_ in low-risk group	*P*
ABT.888	2.22e−16	Bexarotene	4e−05
AICAR	1.3e−05	BMS.754807	3.4e−11
AP.24534	2.5e−08	Bryostatin.1	1.6e−09
ATRA	1.2e−15	CHIR.99021	3.5e−06
AZD.2281	1.7e−07	Cytarabine	1e−05
AZD6244	1.9e−06	FH535	1.5e−05
AZD7762	2.9e−08	FTI.277	*P* < 0.001
AZD8055	1.4e−07	Imatinib	6.6e−08
BMS.708163	1.6e−07	OSI.906	1.9e−13
Bosutinib	1.6e−06	PF.4708671	4.5e−08
BX.795	2e−05	PHA.665752	2.3e−10
Camptothecin	3.3e−08		
CCT007093	4.9e−09		
CCT018159	1.4e−09		
CEP.701	1.5e−05		
CGP.60474	4.8e−06		
CGP.082996	6.1e−06		
CI.1040	9.4e−07		
DMOG	1e−09		
EHT.1864	7e−08		
GDC.0449	1.4e−08		
GDC0941	2.4e−14		
IPA.3	1.9e−06		
JNJ.26854165	5.1e−10		
JNK.9L	3.6e−05		
JNK.Inhibitor.VIII	8.8e−08		
Lenalidomide	*P* < 2.22e−16		
Methotrexate	5.3e−12		
MK.2206	4e−09		
Nilotinib	1.4e−08		
NU.7441	*P* < 0.001		
Nutlin.3a	4.4e−06		
NVP.BEZ235	9e−05		
PD.173074	1.2e−05		
PD.0325901	*P* < 0.001		
PF.02341066	1.3e−05		
RO.3306	2.2e−08		
Rapamycin	*P* < 0.001		
Roscovitine	4.7e−07		
Shikonin	1.5e−06		
Sunitinib	5.6e−07		
Temsirolimus	4.4e−11		
TW.37	3.8e−08		
Vinblastine	2.2e−11		
VX.702	1.6e−07		
X681640	5.2e−10		

## Discussion

Over the past few decades, tumor immunotherapy has achieved remarkable breakthroughs; however, low response rates remain a major challenge due to tumor immune evasion and therapy resistance (Alzeibak et al., 2021; Zhang et al., 2021). ICD transforms immunologically “cold tumors” into inflamed “hot tumors”, elicits T cell-mediated adaptive anti-tumor immunity, and establishes durable immune memory, making it a promising combinatorial strategy to enhance the therapeutic efficacy of ICIs (Kroemer et al., 2013; Yin et al., 2020). Accumulating evidence reveals that chemotherapy-induced ICD transforms immunologically “cold tumors” into “hot tumors” and sensitizes tumors to ICIs. However, merely a fraction of CC patients can respond to immune checkpoint blocking (ICB), highlighting an urgent requirement for predictive biomarkers (Fabian, Wolfson & Hodge, 2021; Galluzzi et al., 2020). Tumor therapy-induced immunogenic cell death reshape the tumor immune microenvironment (Tu et al., 2020; Zhou et al., 2019, 2020). Mechanistically, ICD is accompanied by the exposure and release of many DAMPs, which promote their binding to cognate Pattern Recognition Receptors (PRRs) expressed on PRRs exhibited by innate immune cells, including DCs, macrophages, and monocytes. This process drives the activation, maturation, and migration of these immune cells into lymph nodes bearing tumor antigen-specific cargo. Subsequent presentation of tumor antigens to T cells further enhances immune cell infiltration into the tumor microenvironment (Sprooten et al., 2023; Tu et al., 2020). Currently, two therapeutic agents combined with ICIs have been approved by the FDA. One is BelanCCab Mafodotin, approved by the FDA in 2020 for the treatment of adult patients with relapsed or refractory multiple myeloma (Baines et al., 2022); the other is Rubinetine (Singh et al., 2021), which is used to treat small-cell lung cancer. To date, the objective response rate of ICIs for CC ranged from 4% to 26.3% (Massarelli et al., 2019; Oaknin et al., 2024; Zamarin et al., 2020), with long-term responses achieved in >80% of responding patients (>6 months) (Chung et al., 2019). However, the main limitation of immunotherapy is that it benefits only a small number of patients. Thus, identifying patients who can benefit from immune checkpoint inhibitor therapy will undoubtedly optimize their clinical outcomes and treatment strategies. Given that ICD-related biomarkers hold great potential for improving immunotherapy outcomes, identification of such biomarkers may help optimize treatment stratification for patients with CC. In our study, we demonstrated that the expression of ICD-related genes is closely related to the prognosis of CESC and tumor microenvironment. We demonstrated that the expression of ICD-related genes was closely associated with prognosis and the tumor microenvironment in CESC. Our analysis identified 11 ICD-related genes significantly associated with prognosis in CESC patients, including ATG5, IL1B, NT5E, PDIA3, TNF, CD4, CD8A, CD8B, CXCR3, FOXP3, and IFNG. These identified genes have also been previously reported. ATG5 regulate epithelial mesenchymal transformation of CC and is an indicator of poor prognosis of CC (Zhou et al., 2021). ATG5 alleviates the immunosuppressive tumor microenvironment of CC and inhibits immune escape by enhancing crosstalk between M0 macrophages and initial CD4^+^ T cells (Chen et al., 2023). The number of neoantigens in CC with high expression of CD8A increased (Chen et al., 2017); CD8B is associated with radiotherapy resistance of CC (Feng et al., 2021). CC cells stimulated fibroblasts to express PDL1 through CXCR3 (Chen et al., 2021). IFNG expression level predicts prognosis in patients with CC (Xing, Tian & Jin, 2022). IL1B polymorphism is associated with CC risk (Wagner et al., 2023). NT5E gene encoding CD73 protein, inhibition of CD73 expression can increase the sensitivity of CC cells to cisplatin (Carrera-Martínez et al., 2023). TNF-α expression levels were significantly higher in CC tissues than in normal cervical tissues (Sha et al., 2021). PDIA3 is a marker of poor prognosis of CC, and its expression level is strongly associated with patient survival (Zhang et al., 2022). T cell-intrinsic STING signaling promotes regulatory T cell induction and immunosuppression by upregulating FOXP3 transcription in CC (Ni et al., 2022). We identified two subgroups of ICDs based on the consistent clustering of ICD-related gene expression. High ICD subtypes are associated with favorable clinical outcomes and high levels of immune cell infiltration. In addition, we constructed and validated prognostic risk profiles for six ICD-related genes. This risk model serves as a promising prognostic and immunotherapeutic response predictor for CESC. We further performed external validation in the GEO cohort, confirming its favorable and robust prognostic predictive performance.

Several studies have shown that the TME is a key determinant of tumorigenesis and progression (Binnewies et al., 2018; Kim et al., 2020). Immune cells influence survival rate and response to immunotherapy in patients with CC (Anderson & Simon, 2020; Lei et al., 2020; Mao et al., 2021). Our study showed that patients with a high ICD signature score exhibited significantly higher stromal and immune scores, estimated scores, as well as lower tumor purity, compared with those in the low ICD score group. In addition, we observed significant differences in tumor immune cell infiltration between the high-ICD and low-ICD subgroups. Specifically, the high-ICD group exhibited markedly increased infiltration of CD8^+^ T cells, CD4 memory-activated T cells, follicular helper T (Tfh) cells, M1 macrophages, and resting DCs. In contrast, the low-ICD group presented elevated infiltration of resting CD4 memory T cells, M0 macrophages, DCs, activated mast cells, and eosinophils. In previous reports, a larger lymphocyte population was strongly associated with better prognosis for CC (Christofides et al., 2022). High-density tumor-associated macrophage (TAM) infiltration is a marker of poor prognosis in cancer patients (Christofides et al., 2022; Liu et al., 2020). In this study, the high-ICD group harbored a higher proportion of M1 macrophages, which is consistent with the findings of previous studies. Chemotherapy combined with ICIs can improve the therapeutic effect of CC patients (Tewari et al., 2024). It has now been shown that PD-1 and CTLA4 inhibitors can leverage the host immune system to achieve antitumor effects in CC therapy (Borcoman & Le Tourneau, 2020). Screening appropriate candidates for ICB therapy is of great clinical significance in routine management. We analyzed the differences in PD-1 and CTLA-4-related IPS across LASSO-defined risk subgroups. Higher IPS were observed in the low-risk cohort, indicating that low-risk patients are more responsive to ICIs treatment. Additionally, high-risk patients exhibited higher TIDE scores, indicating that this subgroup was more prone to immune escape. ICD resistance refers to the failure of cancer cells to undergo immunogenic cell death upon stimulation, resulting in impaired anti-tumor immunity. Key mechanisms include epigenetic silencing of RIPK3/MLKL, defective ER stress–DAMP signaling, hypoxic/immunosuppressive tumor microenvironment, and hyperactive autophagy or antioxidant systems. Such resistance maintains “cold tumors” and blunts responses to chemo-, radio-, photodynamic and immune checkpoint therapies. Strategies to reverse ICD resistance include epigenetic modifiers, nanocarrier-mediated ICD inducers, metabolic modulation, and combined immune checkpoint blockade to restore DAMPs release and DC-T cell activation (Barjij & Meliani, 2025; Liang et al., 2026; Papavassiliou et al., 2026). This study has several limitations. First, we only adopted ICD-related gene sets retrieved from published studies, and the number of normal cervical samples in the TCGA database was relatively limited. Second, the specific biological functions and mechanisms of these ICD signature genes were not further explored. Third, additional validation using clinical CESC tissues and cell lines is needed to confirm the clinical implications of ICD signature gene expression in CESC. Moreover, differential infiltration of immune cells in the tumor microenvironment does not directly confirm their interactive relationships. Further in-depth mechanistic investigations are therefore required to fully verify and supplement our findings.

## Conclusions

In this study, we identified eleven ICD-related genes as independent prognostic biomarkers for CC, including ATG5, IL1B, NT5E, PDIA3, TNF, CD4, CD8A, CD8B, CXCR3, FOXP3 and IFNG. We further established a novel molecular classification system for CC and comprehensively analyzed its functional enrichment, immune features, prognostic value and therapeutic implications. Collectively, this study offers novel insights and a promising theoretical reference for immunotherapy in CC patients.

## Supplemental Information

10.7717/peerj.21635/supp-1 Supplemental Information 1 Code.

10.7717/peerj.21635/supp-2 Supplemental Information 2 Differential expression of ICD-related genes in 306 patients with CC and 3 normal cervical individuals.

10.7717/peerj.21635/supp-3 Supplemental Information 3 ICD-related genes that were used as initial filtering.

## Abbreviations

AIC: Akaike information criterion
CC: cervical cancer
CESC: cervical squamous cell carcinoma and endocervical adenocarcinoma
CRT: calreticulin
CTLA-4: cytotoxic T-lymphocyte-associated protein-4
DAMPs: damage associated molecular patterns
DEGs: Differentially expressed genes
FDA: the Food and Drug Administration
FDR: false discovery rate
GO: Gene Ontology
GSEA: Gene set enrichment analysis
HMGB1: high mobility group box 1
IC_50_: the half maximal inhibitory concentration
ICB: immune checkpoint blocking
ICD: Immunogenic cell death
IHC: immunohistochemistry
IPS: Immunophenoscore
KEGG: Kyoto Encyclopedia of Genes and Genomes
LASSO: least absolute shrinkage and selection operator
MAF: Minor Allele Frequency
PD-1: programmed cell death protein-1
PD-L1: programmed cell death ligand 1
PRRs: Pattern Recognition Receptors
RCD: regulated cell death
ROC: receiver operating characteristic
SNV: standard normalized variate
SNVs: MAF-type single nucleotide variants
TAM: tumor-associated macrophage
TCGA: The Cancer Genome Atlas
TFH: follicular helper T cells
TIDE: tumor immune dysfunction and exclusion
TMB: tumor mutational burden
TME: Tumor microenvironment
Tregs: regulatory T cells

## Additional Information and Declarations

### Data Availability

The following information was supplied regarding data availability:

The source code is available in the Supplemental Files.

TCGA, GEO, TCIA and TIDE datasets are available at Zenodo:

- Li, X. (2026). Prediction of prognosis and treatment effect of cervical cancer by immunogenic cell death-related genes [Data set]. Zenodo. https://doi.org/10.5281/zenodo.21805726.
