# Supplementary material for: Prediction of prognosis and treatment effect of cervical cancer by immunogenic cell death-related genes
Source: PeerJ. 2026 Oct 7;14:e21635. doi: 10.7717/peerj.21635 (PMC13653537; doi:10.7717/peerj.21635)
Supplement: Supplementary file 1 [file peerj-14-21635-s002.docx]

| gene | Mean of the normal group | Mean of the tumor group | logFC | *p* |
| --- | --- | --- | --- | --- |
| BAX | 50.63023 | 105.9799 | 1.065719 | 0.005182 |
| CALR | 586.9237 | 1235.168 | 1.073463 | 0.008622 |
| CASP8 | 6.6765 | 13.90348 | 1.058282 | 0.033991 |
| ENTPD1 | 20.67077 | 5.257813 | -1.97506 | 0.00344 |
| FOXP3 | 0.737167 | 5.566404 | 2.916683 | 0.004977 |
| HSP90AA1 | 434.5558 | 892.0101 | 1.037519 | 0.017014 |
| IFNG | 0.155 | 3.653098 | 4.55878 | 0.018902 |
| IL10 | 0.142133 | 0.67735 | 2.252656 | 0.023634 |
| IL1R1 | 67.80693 | 20.82238 | -1.7033 | 0.008788 |
| LY96 | 50.0873 | 19.26226 | -1.37867 | 0.008622 |
| MYD88 | 24.89747 | 56.66124 | 1.186363 | 0.013727 |
| NT5E | 86.03767 | 21.39198 | -2.0079 | 0.008622 |
| TLR4 | 7.5597 | 1.836037 | -2.04173 | 0.005287 |
| TNF | 0.839333 | 8.991997 | 3.421326 | 0.012532 |
