# Supplementary material for: Prediction of prognosis and treatment effect of cervical cancer by immunogenic cell death-related genes
Source: PeerJ. 2026 Oct 7;14:e21635. doi: 10.7717/peerj.21635 (PMC13653537; doi:10.7717/peerj.21635)
Supplement: Supplementary file 2 [file peerj-14-21635-s003.docx]

ATG5

BAX

CALR

CASP1

CASP8

CD4

CD8A

CD8B

CXCR3

EIF2AK3

ENTPD1

FOXP3

HMGB1

HSP90AA1

IFNA1

IFNB1

IFNG

IFNGR1

IL10

IL17A

IL17RA

IL1B

IL1R1

IL6

LY96

MYD88

NLRP3

NT5E

P2RX7

PDIA3

PIK3CA

PRF1

TLR4

TNF
